# Ring-Enhancing Progressive Multifocal Leukoencephalopathy Mimicking Glioma in a Presumed Immunocompetent Patient With a History of Multiple Sclerosis: A Case Report and Review of the Literature

**DOI:** 10.7759/cureus.45543

**Published:** 2023-09-19

**Authors:** Sarah-Marie C Gonzalez, Anthony Nguyen, Jose M Soto, Yuan Shan

**Affiliations:** 1 Neurosurgery, Baylor Scott & White Medical Center - Temple, Temple, USA; 2 Pathology, Baylor Scott & White Medical Center - Temple, Temple, USA

**Keywords:** primary brain tumors, human polyomavirus 2, ring enhancing lesions, multiple sclerosis, progressive multifocal leukoencephalopathy

## Abstract

The differential diagnoses of ring-enhancing lesions of the brain parenchyma is broad, but complete ring-enhancing lesions often indicate a neoplastic or infectious process. We present a case of a 70-year-old female with a history of multiple sclerosis (MS) who was not on current disease-modifying therapy (DMT) and was found to have a ring-enhancing lesion that mimicked a high-grade glioma. The patient underwent gross total resection, and histopathologic and molecular analysis revealed a diagnosis of progressive multifocal leukoencephalopathy (PML). A subsequent medical workup on the patient was unrevealing aside from mild lymphopenia. This is a unique case that highlights both an unusual clinical presentation and radiographic appearance of PML. There is a known associated increased risk of PML with the use of some DMTs for MS. However, this case raises the question of the possibility of developing PML years after interferon beta-1a therapy in a patient without overt immunosuppression.

## Introduction

Ring-enhancing lesions of the brain parenchyma on magnetic resonance imaging (MRI) have broad differential diagnoses, including abscesses, metastases, high-grade gliomas, and vascular, infectious, or inflammatory processes [1]. Multiple sclerosis (MS) is an inflammatory demyelinating disease of the central nervous system, which can infrequently exhibit nodular or ring-like contrast enhancement, and glial tumors have been known to coexist with demyelinating MS lesions [2-3]. Progressive multifocal leukoencephalopathy (PML) is an infection of the glia with human polyomavirus 2, commonly referred to as the John Cunningham (JC) virus of the Papovaviridae family, which occurs in immunocompromised individuals [4]. PML can also present as ring-enhancing lesions on MRI. The JC virus is gliotropic and will infect both astrocytes and oligodendrocytes. Infection of the latter leads to demyelination [5]. The typical astrocyte structure is disrupted, and the nuclei become pleomorphic, akin to findings of malignant gliomas. These astrocytes may stain positive for p53 on immunohistochemistry, further mimicking malignant gliomas [4]. The diagnosis of PML is therefore confirmed via additional identification of enlarged basophilic oligodendrocytes and if the JC virus can be detected by in situ DNA hybridization with the JC probe or by immunohistochemical stain if anti-JC virus antibodies are available [4].

## Case presentation

**Figure 1:**
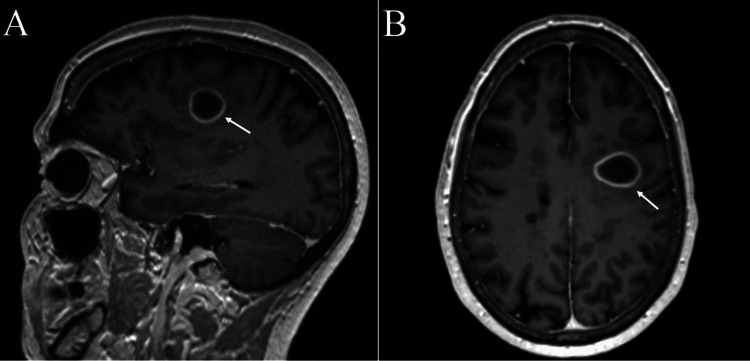
MRI brain Magnetic resonance imaging (MRI) of the brain with T1 post-contrast sagittal (A) and axial (B) slices demonstrating a 2.1 cm x 2.6 cm x 1.9 cm ring-enhancing lesion within the left frontal centrum semiovale superior to the frontal operculum, as indicated by the white arrows.

A 70-year-old female with history of multiple sclerosis presented to a neurosurgery clinic for evaluation of a 2.1 cm ring-enhancing mass within the left frontal centrum semiovale, superior to the frontal operculum (Figure 1) demonstrated on an MRI of her brain. The patient reported progressively worsening word-finding difficulty ongoing for the past several weeks, prompting evaluation with the brain MRI. The patient denied any issues with language comprehension. She had a history of multiple sclerosis and had been on interferon beta-1a therapy previously, but due to disease stability and absence of symptoms, this was stopped approximately 11 years prior. Her last symptomatic relapse was in 2004, during which she experienced transient left hemiparesis and difficulty with stereognosis. Her last brain MRI was performed three years prior and did not demonstrate the aforementioned lesion. On physical examination, she had no focal neurologic deficits. She did not exhibit signs of aphasia at this visit. Given that the appearance of the lesion included a differential diagnosis of a high-grade glial neoplasm, both biopsy and resection were offered to the patient, and she opted to proceed with surgical resection with the understanding that her language function would likely worsen.

**Figure 2:**
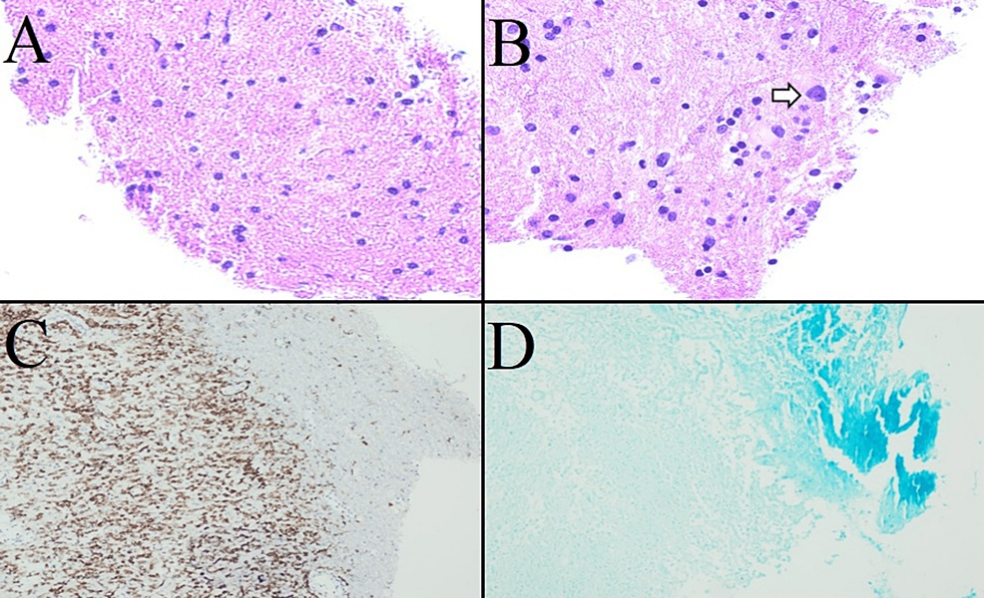
Histopathology (A) Moderately hypercellular specimen visible with hematoxylin and eosin (H&E) stain under 200x magnification. (B) H&E stain under 400x magnification demonstrating a large oligodendroglial cell (white arrow) with an enlarged nucleus, which is an infected cell suggestive of progressive multifocal leukoencephalopathy. (C) Cluster of differentiation 68 stain with prominent positivity, demonstrating macrophages, suggestive of a reactive process. (D) Myelin Luxol fast blue stain demonstrates loss of myelin within the lesion compared to the peripheral internal control.

Stereotactic neuronavigation was utilized to map out the entry and plan the operative trajectory, and a craniotomy was performed in a standard fashion. The lesion was approached via a transfrontal, transcortical approach. The lesion appeared abnormal and grayish and was resected in a piecemeal fashion. There were no intra-operative complications. The patient was taken to the intensive care unit postoperatively and had difficulty with word retrieval, mimicking mild expressive aphasia. She otherwise recovered uneventfully and was discharged to an inpatient rehabilitation facility. Histopathology demonstrated enlarged oligodendrocytes with large, smashed-appearing nuclei suggestive of a diagnosis of PML (Figure 2). Molecular analysis with polymerase chain reaction (PCR) was positive for the JC virus, confirming the diagnosis of PML. Subsequent serum HIV testing was negative. She returned to clinic one month later and reported that her expressive aphasia was improving.

## Discussion

This patient’s clinical course highlights an unusual case of a patient with MS presenting with a solitary complete ring-enhancing lesion suspicious for glial neoplasm that turned out to be PML. A prior report by Kleinschmidt-DeMasters and Tyler suggested a link between MS treatments, specifically natalizumab and interferon beta-1a, with PML [6]. With the introduction of DMTs, reactivation of viruses is increasingly demonstrated among the multiple sclerosis population, although this patient had been off of DMTs for 11 years, and she had only been on interferon beta. Although natalizumab, dimethyl fumarate, fingolimod, and ocrelizumab are well described to have associations with PML, subsequent studies have suggested that interferon beta has only been associated with PML when in combination with natalizumab or immunosuppression [7-9].

Sriwastava et al. presented a case report of a patient diagnosed with PML in the setting of no immunosuppression disorder but a recent COVID-19 infection [10]. Their report included a literature review that yielded an additional 21 cases of PML in immunocompetent patients with no underlying immunosuppressive condition. Classic MRI findings consistent with PML, such as T2 periventricular and/or subcortical involvement hyperintensities, without T1 gadolinium enhancement, were demonstrated in 21 of the 22 patients [10,11]. Only one of the 22 patients had MRI findings with enhancing lesions; similar to our patient, one of the lesions was cystic in appearance and demonstrated ring enhancement. Given the atypical MRI findings, the diagnosis of PML was not realized until brain autopsy. Lehmann et al. reported a case of a patient whose hypogammaglobulinemia was recognized after diagnosis of PML, leading to a diagnosis of common variable immunodeficiency syndrome (CVID) [9]. In the case of our patient, no immunosuppressive condition has been formally diagnosed, but she was noted to have a mild lymphopenia during her admission, raising the possibility of an undiagnosed CVID.

This case thus highlights two interesting points: The first is the differential diagnosis of a ring-enhancing lesion. The diagnosis of a complete ring-enhancing lesion in a patient with a history of MS treated only with interferon beta-1a would most likely be an atypical MS lesion or a glioma, but the diagnosis in our patient’s case was ultimately PML. In addition, although interferon beta-1a has only been associated with the development of PML when utilized in combination with natalizumab, our patient has no other risk factors for PML aside from lymphopenia [6]. Patients with a history of MS previously on interferon beta-1a who present with a ring-enhancing lesion suspicious for a glial neoplasm may benefit from a thorough immunodeficiency work-up in addition to HIV testing. If such a work-up is unrevealing, these patients may benefit from a lumbar puncture with PCR for the JC virus. If PCR is negative, consideration of a brain biopsy may be appropriate prior to full resection.

## Conclusions

The differential diagnosis of a ring-enhancing lesion on MRI is broad, but the mainstay of treatment varies greatly depending on the diagnosis. We presented herein the case of a patient with MS whose ring-enhancing lesion was suspicious for a neoplastic process. Histopathologic and molecular analyses subsequently determined that the lesion represented PML, although she had no known true risk factors. Although clinical history can help to narrow the differential diagnosis, a history of interferon beta-1a use should prompt the consideration of atypical diagnoses, and further research into interferon beta-1a and progressive multifocal leukoencephalopathy should be considered.
